# Why Growing Retractions Are (Mostly) a Good Sign

**DOI:** 10.1371/journal.pmed.1001563

**Published:** 2013-12-03

**Authors:** Daniele Fanelli

**Affiliations:** Science, Technology and Innovation Studies, The University of Edinburgh, Edinburgh, United Kingdom

## Abstract

In a new Essay in the Research Integrity Series, Daniele Fanelli examines the evidence and possible reasons for the rising number of retractions.

*Please see later in the article for the Editors' Summary*

**Summary Points**

- Corrections to scientific papers have been published for much longer than retractions, and show little sign of a recent increase.
- The number of journals issuing retractions has grown dramatically in recent years, but the number of retractions per retracting-journal has not increased.
- The number of queries and allegations made to the US Office of Research Integrity has grown, but the frequency of its findings of misconduct has not increased.
- Therefore, the rising number of retractions is most likely to be caused by a growing propensity to retract flawed and fraudulent papers, and there is little evidence of an increase in the prevalence of misconduct.
- Statistics on retractions and findings of misconduct are best used to make inferences about weaknesses in the system of scientific self-correction.

**Research Integrity Series**

This is one article in an occasional *PLOS Medicine* series on research integrity that examines issues affecting the ethics of health research worldwide.

## Introduction

Retractions of scientific papers have recently been in the spotlight. Unfortunately, the interpretation of statistics about them is often flawed. The realisation that most retractions follow from scientific misconduct [1] seems to have reinforced, in the minds of both scientists and journalists, the idea that data on retractions, and generally data on findings of misconduct, provide information about the prevalence of fraud itself [2]. The recent growth in retractions, for example, is often invoked as evidence that scientific misconduct is increasing [2]–[4]. Similarly, findings that more papers are retracted by high-ranking journals, in biomedical fields, and in certain countries, and that more men than women are found guilty of misconduct are used to suggest possible risk factors for scientific misbehaviour [5]–[9]. The obvious alternative interpretation—that these statistics are proportional not to the prevalence of misconduct but to the efficiency of the system that detects it—is given equal or lower attention [3],[4],[9]–[11].

I will present four lines of evidence to suggest that retractions have grown not because of rising misconduct—an explanation that I call the “growing misconduct” hypothesis (GMH)—but because scientists have become more aware of and responsive against fraudulent and flawed research. I call this second explanation the “stronger system” hypothesis (SSH), although this is partially a misnomer, because a recent strengthening of measures against misconduct is not just a hypothesis, but a historical fact (Box 1).

**Box 1. Systems to Fight Scientific Misconduct Are Recent, and Growing**

- The world's first legal definition of scientific misconduct and the first national office of scientific integrity were only established in the late 1980s, in the United States [24].
- To this day, most countries, even in Europe, lack national frameworks to deal with allegations of misconduct [25].
- Few universities currently provide research integrity courses; these courses differ widely in content, and evidence about their effectiveness is inconclusive [26].
- Editors and authors have recently acquired an unprecedented ability to detect all forms of plagiarism, thanks to online tools [27].
- Most journals still lack guidelines and clear policies on how to deal with misconduct and retraction [22],[23].

The data I present in this essay to support my argument were retrieved from the Thomson Reuters Web of Science (WoS) database, which is unique in covering over a century of publications. The WoS database marks both errata (corrections to previous papers) and retractions as “correction” or “correction, addition” (total *n*=304,000 circa). Retractions can be retrieved from all these “corrections” by selecting those that include the term “retraction” in their title (total *n*=2,294). Notably, most previous studies on retractions have used the PubMed database. Unlike WoS, PubMed has a specific category for retractions. However, PubMed restricts its coverage mostly to biomedical research and only started recording errata in 1987. This limitation may have caused some of the misunderstandings that this essay aims to debunk.

## Errata Have Not Increased in Frequency

**Figure 1:**
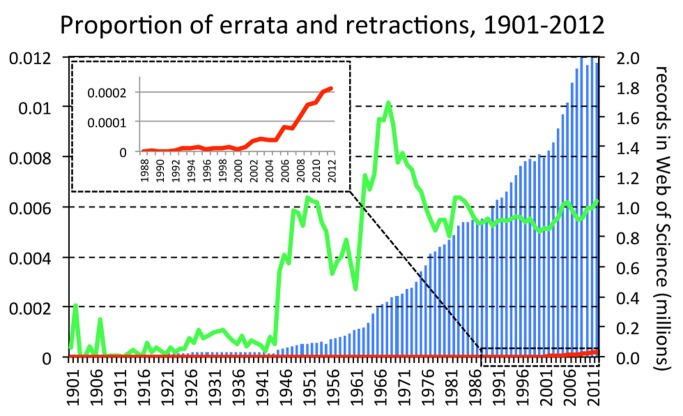
Proportion of errata and retractions amongst all records in the Web of Science database, by year. Bars represent total number of records added each year to the Web of Science database. Green, proportion of Web of Science records marked as “correction” or “correction, addition,” excluding those with “retraction” in the title. Red, proportion of those that have “retraction” in the title.

As observed in previous studies that used PubMed, the number of retractions in the WoS database has grown dramatically over the last 20 years (Figure 1). Although the first retraction recorded in WoS is more recent than that recorded in PubMed (1989 versus 1977), the picture is substantially the same. However, unlike PubMed, the WoS database shows that errata have been published since at least 1901. This should come as no surprise. The publication of errata predates the invention of the printing press [12], and has always been an option available to scientists and editors. What is remarkable, however, is that, despite a steady increase in the number of publications covered by the WoS, the proportion of errata has remained relatively constant since the 1970s (and arguably since the 1950s). The scarcity of errata prior to 1945 could partly be an artefact created by limitations in the database (for example, in the coverage of older literature from the humanities and social sciences). Nonetheless, there seems to have been little change in the relative abundance of errata across disciplines. Errata have been and still are published most frequently in the WoS Research Areas of Physics (*n*=38,899, starting in year 1905), Chemistry (*n*=31,463, 1911), Engineering (*n*=22,521, 1927), Biochemistry and Molecular Biology (*n*=21,155, 1930), and General Internal Medicine (*n*=16,443, 1901).

Taking the year 1980 as the starting point of a recent, and therefore most reliable, data series, the frequency of retractions has grown 20% per year, whilst that of errata has not grown to any significant extent (for retractions, b=0.21±0.009 SE, t=23.1, *p*<0.001; for errata, b=0.002±0.001 SE, t=1.2, *p*=0.228; unless otherwise stated, analyses in this essay employ a generalized linear model assuming quasi-Poisson distribution of errors and log link function). This lack of growth in errata is the first line of evidence that favours the SSH. Survey data suggest that misconduct lies at the extreme of a continuum of “sloppy” and questionable research practices [13]. If the recent growth of retractions were being driven by an increasing propensity of researchers to “cut corners,” we would expect minor infractions, and therefore the frequency of published errata, to increase just as fast as, if not faster than that of retractions.

The content of correction notes was not examined in the present analysis, so the possibility that the nature of errors being corrected could have changed over the years cannot be excluded. Evidence that errors have become more serious could partially support the GMH, but could also indicate that proactive responses towards more serious mistakes have increased, which would support the SSH. Evidence that the average seriousness of errors has decreased, on the other hand, would suggest that the same flaws that were previously only corrected now lead to a full retraction, again supporting the SSH.

## The Proportion of Journals Retracting Articles Has Increased

**Figure 2:**
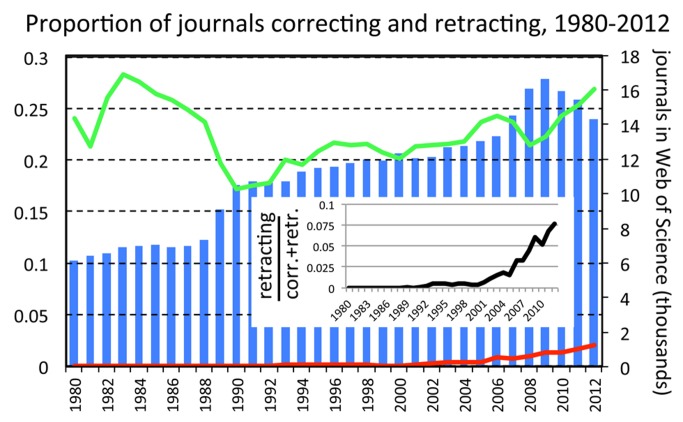
Proportion of journals issuing corrections or retractions amongst all journals covered by the Web of Science database, by year. Bars represent total number of journals covered by the Web of Science database each year. Green, proportion of journals that published at least one record marked as “correction” or “correction, addition,” amongst all journals appearing in the Web of Science database. Red, proportion of these that published at least one correction with “retraction” in the title. Black, proportion of retracting-journals amongst all those correcting or retracting.

If the SSH is correct, retractions would be growing because more journal editors are prepared to retract papers. If this is the case, then the proportion of journals retracting papers, but not the proportion of journals correcting papers, should grow. This is indeed what is occurring (Figure 2). The proportion of journals issuing corrections was highest in the early 1980s, although this could be an artefact caused by markedly fewer journals being recorded in the WoS database before 1990. Between 1992 and 2012, the proportion of correcting-journals shows a moderate growth (b=0.009±0.002SE, t=5.0, *p*<0.001), but this is nearly 25 times slower than the growth in the proportion of retracting-journals (b=0.209±0.011 SE, t=17.9, *p*<0.001). This analysis cannot exclude the possibility that the rise in retracting-journals is caused by misconduct being reported in specialties that previously saw none. However, the SSH would provide a simpler explanation for this phenomenon (researchers and editors in these specialties have become more proactive), than the GMH (misconduct has spread into specialties that used to be “pristine”).

## The Number of Retractions per Retracting-Journal Has Not Increased

**Figure 3:**
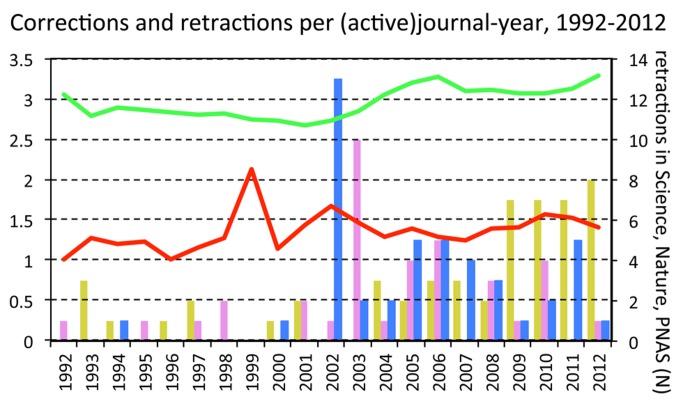
Corrections per-correcting-journal, retractions per-retracting-journal, and number of retractions issued by three major journals, by year. Bars represent number of papers retracted by the journals *Science* (pink), *Nature* (blue), and *PNAS* (yellow). Green line, corrections per-correcting-journal (i.e., total number of Web of Science records marked as “correction” or “correction, addition,” divided by total number of journals that issued at least one item of that type). Red line, retractions per-retracting-journal (i.e., same as for green line, but for corrections that have “retraction” in title).

If there is a link between the growing numbers of retractions and growing misconduct as the GMH proposes, the number of retractions should have risen independently of the number of journals that are active in retracting papers. A sense of historical trends can be gained by examining the WoS records of three journals that have retracted substantial numbers of papers since 1992, namely *Nature*, *Science*, and *PNAS*. Only *PNAS* shows an increase in retractions in recent years (Figure 3). Retractions in the other two journals peaked in 2002–2003—in both cases because of multiple retractions related to the Jan Hendrik Schön affair (nine in *Science* in 2002 and seven in *Nature* in 2003) [14]. Even if these peaks are ignored, there is no evidence of a gradual increase in retractions in any of these journals. Instead, an abrupt rise in retractions occurred in the last decade in all three, as might be expected if editorial policies and behaviours had suddenly changed.

A less direct, but more powerful test to distinguish between the GMH and the SSH comes from dividing the number of retractions by the number of retracting-journals. According to the GMH, if misconduct cases were growing, each journal should be dealing, on average, with a growing number of retractions. Interestingly, there is indeed an increase in retractions per-retracting-journal, but it is very modest (Figure 3; b=0.009±0.005 SE, t=1.9, *p*=0.071), of a similar magnitude to that observed amongst corrections (b=0.007±0.002 SE, t=4.3, *p*<0.001).

Could this small (less than 1%) yearly increase in retractions and corrections per-(active)-journal be the first unequivocal symptom of rising fraud and sloppiness? Unfortunately, other confounding variables remain to be dealt with. A relevant source of error, which for brevity was ignored in this analysis, is represented by “prolific” fraudsters. The rate of multiple retractions caused by single individuals has grown, and this significantly skews all estimations [8]. Even more important is the fact that retractions due to misconduct are just the terminal phase of a long process. This process usually starts with an allegation made to an institutional authority, which should examine the case and eventually lead an investigation. If unequivocal evidence of misconduct is found, editors of journals that published fraudulent material should be informed, in order for them to take action in ways that they deem appropriate. Therefore, a central prediction of the GMH is that the rise in retractions should be paralleled by a rise in findings of misconduct. Is this the case?

## Findings of Misconduct by the US Office of Research Integrity Have Not Increased

**Figure 4:**
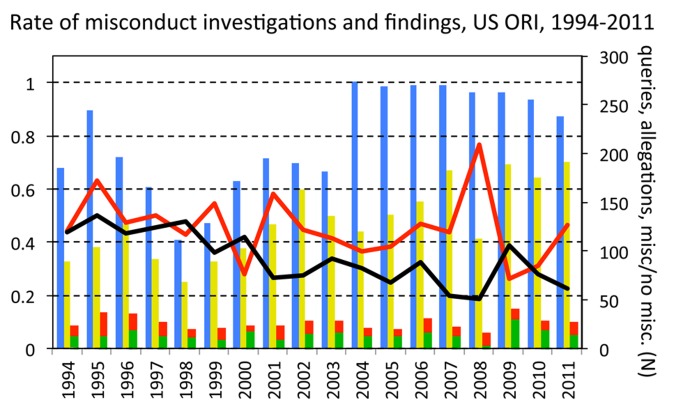
Queries, allegations, investigations, and findings of scientific misconduct made at the United States Office of Research Integrity, by year. Bars represent numbers of queries (blue), allegations of misconduct (yellow), and investigations closed with either a finding of misconduct or no misconduct (red and green, respectively). Black line, number of closed investigations divided by number of allegations. Red line, proportion of investigations closed with a finding of scientific misconduct.

Between 1994 and 2011 (the period for which reliable data are available), the number of queries and actual allegations of misconduct made to the US Office of Research Integrity (ORI) nearly doubled, as would be expected if researchers in the US have become more aware of and more proactive about misconduct (Figure 4). The number of actual investigations, however, has not increased significantly (b=0.483±0.333 SE, t=1.4, *p*=0.164). Indeed, compared to the number of new allegations made each year, the number of closed (completed) investigations has tended to decrease (b=−0.027±0.015 SE, t=−1.8, *p*=0.082). This decrease could be an artefact, caused by limits to the number of cases that ORI is able to process each year [15]. Most crucially, however, the proportion of ORI investigations that were concluded with a finding of misconduct has also not increased, and if anything it shows signs of decreasing (generalized linear model assuming quasi-binomial errors and logit link function, b=−0.008±0.005 SE, t=−1.638, *p*=0.121).

## Discussion

Data from the WoS database and the ORI offer strong evidence that researchers and journal editors have become more aware of and more proactive about scientific misconduct, and provide no evidence that recorded cases of fraud are increasing, at least amongst US federally funded research. The recent rise in retractions, therefore, is most plausibly the effect of growing scientific integrity, rather than growing scientific misconduct.

In general, statistics about misconduct findings and retractions appear to be most economically and most usefully interpreted as reflecting strengths and weaknesses in the systems of detection and correction. For example, a recent study reported that male researchers, particularly faculty members, are overrepresented amongst individuals found guilty of misconduct by the ORI, compared to average sex ratios in the life sciences [6]. This finding was discussed almost exclusively as suggesting a psychological predisposition of males toward scientific misconduct. However, alternative interpretations are plausible and should be examined more carefully [16]. For example, females could be less likely to get caught, and more effective in apologizing and negotiating their defence. Men, on the other hand, are assumed to be prone to risk and crime, which may facilitate allegations and negative judgments. Moreover, male faculty hold larger and more numerous grants, which gives them more opportunities to commit misconduct. Finally, ORI investigations are limited to research funded by the US Department of Health and Human Services, a sample that may have a different sex ratio to the rest of the life sciences.

Even if more obvious (and therefore easily detectable) forms of misconduct turn out to be stable or declining, bias and subtle forms of falsification might still be on the rise (Box 2). Ironically, this would be a consequence of the growing awareness of misconduct suggested by my analyses. Rather like professional athletes, who strive to maximize performance-enhancing practices within the allowed limits, scientists might be getting better at “pushing” their findings in the desired direction and stopping right before the “misconduct threshold” or at avoiding getting caught if they trespass it. Survey data support this scenario, by showing that, while researchers have become less likely to admit having committed scientific misconduct, they are not less likely to report having observed it amongst colleagues [13].

**Box 2. Is Scientific Misconduct Increasing?**

There is no conclusive evidence that the prevalence of scientific misconduct is higher today than in the past. However, the scientific profession is changing in ways that, growing evidence suggests, could increase the expression of unconscious biases, questionable practices, and possibly misconduct.

- Competition for limited academic resources is likely to keep growing [28], and performance-evaluation metrics are increasingly influencing careers and funding allocation worldwide [29].
- Academics report suffering personal and institutional pressure to publish, particularly in the United States [30].
- National pressures and performance-based incentives correlate with submission rates to high-ranking journals, but not with subsequent acceptance rates [31].
- The proportion of reported negative results and statistically non-significant results has decreased over the years in most biological and social disciplines [32],[33], and is inversely correlated to academic productivity across the United States [34].
- Studies are more likely to report extreme effects supporting the experimental hypothesis when their corresponding author is based in the United States rather than in Europe, at least in economics and the behavioural sciences [35]–[37].

If the above scenario corresponds to reality, then new challenges lie ahead. Part of the effort currently devoted to fighting obviously fraudulent behaviours might need to shift towards the “grey area” of questionable research practices. These practices cannot easily be detected or punished, but can be kept at bay by ensuring that research findings are reproduced, replicated, and evaluated critically. This process of self-correction is facilitated by transparent reporting practices [17],[18] and would have its ultimate manifestation in the un-embarrassed correction and retraction of flawed papers.

An unjustified stigma currently surrounds retractions, and the opaqueness of many retraction notices betrays improper feelings of embarrassment [1]. Nearly 60% of retraction notices linked to misconduct only mention error, loss of data or replication failure, and less than one-third point to a specific ethical problem [19]. Editors writing these notices often use ambiguous euphemisms in place of technical definitions of misconduct, perhaps to prevent legal actions (see retractionwatch.wordpress.com). Although retraction notices are becoming more transparent, many journals still lack clear policies for misconduct and retraction, and existing policies are applied inconsistently [19],[20],[21]. It is worth pointing out that journals with a high impact factor are more likely to have clear policies for scientific misconduct [22],[23]. This datum offers a simple, and largely overlooked, explanation for the correlation observed between journal impact factor and retraction frequency, which instead is usually attributed to higher scrutiny and higher prevalence of fraudulent papers in top journals [1],[7].

## Conclusions

Growing numbers of retractions are most plausibly a sign that researchers and journal editors are getting better at identifying and removing papers that are either fraudulent or plainly wrong. These extremely positive changes need to be promoted further because, although the exact prevalence of flawed and manipulated studies is unknown, it is almost certainly higher than the current rate of retractions [13]. By incorrectly equating the prevalence of retractions with that of misconduct, the scientific community risks hindering this positive trend. Editors and authors who proactively remove flawed publications from the literature should be rewarded for their integrity and held up as examples. Conversely, we should be highly critical and suspicious of those journals and fields in which papers are retracted very rarely, if at all.
